# Intraoral desmoplastic melanoma requiring radical multidisciplinary resection and free flap reconstruction: case report and literature review

**DOI:** 10.1093/jscr/rjag498

**Published:** 2026-09-01

**Authors:** Frank A Álvarez Vásquez, Juan P Zaraza Duarte, Laura A Garzón Bautista, Iván E Rodríguez Mantilla, Nicol D Cala Gómez, Daniela Marulanda Grajales, Nidya C Guataquira Sierra

**Affiliations:** School of Medicine and Health Sciences, Universidad del Rosario, Calle 12C No. 6-25, Bogotá, D.C. 110010, Colombia; Specialization in Epidemiology, Faculty of Health Sciences, Universidad Autónoma de Bucaramanga (UNAB), Avenida 42 No. 48-11, Bucaramanga, Santander 680003, Colombia; School of Medicine and Health Sciences, Universidad del Rosario, Calle 12C No. 6-25, Bogotá, D.C. 110010, Colombia; Fundación Universitaria Sanitas, Northern Campus, Avenida Carrera 7 No. 173-64, Bogotá, D.C., Colombia; Department of Plastic and Reconstructive Surgery, Hospital de San José, Fundación Universitaria de Ciencias de la Salud, Carrera 19 No. 8A-32, Bogotá, D.C., Colombia; Department of Plastic Surgery, Faculty of Medicine, Universidad Nacional de Colombia, Avenida Carrera 30 No. 45-03, Ciudad Universitaria, Bogotá, D.C., Colombia; Department of Oral and Maxillofacial Surgery, Hospital Militar Central, Transversal 3C No. 49-02, Bogotá, D.C., 110231, Colombia; Department of Head and Neck Surgery, Hospital Universitario Mayor Méderi, Calle 24 No. 29-45, Bogotá, D.C., Colombia

**Keywords:** desmoplastic melanoma, oral cavity neoplasms, melanoma, mucosal, head and neck neoplasms

## Abstract

Desmoplastic melanoma (DM) is a rare variant of malignant melanoma characterized by spindle cell proliferation within dense collagenous stroma and atypical immunohistochemical profile. Intraoral involvement is exceedingly uncommon and poses diagnostic and therapeutic challenges. We report the case of a male patient in his fourth decade presenting with enlarging right orofacial mass associated with extensive soft tissue and osseous infiltration. Histopathological evaluation revealed infiltrative spindle cell neoplasm with focal melanin pigment and limited expression of conventional melanocytic markers, requiring integrated diagnostic approach. The patient underwent radical en bloc resection followed by immediate reconstruction using anterolateral thigh free flap. Adjuvant systemic therapy was not administered; however, it was evaluated within multidisciplinary tumor board. At 12-month follow-up, no locoregional recurrence was observed. This case underscores diagnostic complexity of intraoral DM and highlights importance of multidisciplinary oncologic approach in optimizing management outcome.

## Introduction

Desmoplastic melanoma (DM) is an uncommon histologic variant of melanoma characterized by proliferation of spindle-shaped melanocytes within a dense collagenous stroma [1]. It most frequently arises in chronically sun-exposed regions of the head and neck and accounts for a small proportion of all melanomas [1, 2]. In contrast, mucosal desmoplastic melanoma, particularly involving the oral cavity, is exceptionally rare and remains poorly characterized in the literature [2, 3].

Clinically, desmoplastic melanoma often presents as a firm, indurated, and frequently amelanotic lesion that may mimic benign fibrous proliferations or other spindle cell neoplasms [3]. This atypical presentation contributes to delayed diagnosis and allows for significant local progression prior to treatment. Within the oral cavity, this diagnostic difficulty is compounded by the complex anatomy and the broad differential diagnosis, including sarcomas, neural tumors, and spindle cell carcinomas [4, 5].

Histopathologically, desmoplastic melanoma demonstrates considerable immunophenotypic variability. Although S100 and SOX10 are typically expressed, staining may be weak or focal, while conventional melanocytic markers such as HMB-45 and Melan-A are often absent [4, 6]. This variability necessitates a comprehensive diagnostic approach integrating morphology, immunohistochemistry, and, when available, molecular analysis [7].

Surgical resection with adequate oncologic margins remains the cornerstone of treatment [1, 3]. However, the infiltrative growth pattern and frequent perineural invasion make margin control particularly challenging in anatomically complex regions such as the oral cavity [7]. As a result, radical composite resections are often required, resulting in significant functional and esthetic deficits.

Immediate microvascular reconstruction has become essential to restore speech, swallowing, and facial contour. The anterolateral thigh (ALT) free flap offers important advantages, including large tissue volume, long vascular pedicle, and versatility for three-dimensional reconstruction [8].

Contemporary management of mucosal melanoma has evolved toward a multimodal paradigm. Adjuvant systemic therapy, particularly immune checkpoint inhibition targeting the PD-1 pathway, is currently recommended in resected high-risk melanoma, although its benefit in mucosal subtypes remains less pronounced than in cutaneous disease [9]. Additionally, adjuvant radiotherapy has been proposed in desmoplastic melanoma due to evidence suggesting relative radiosensitivity and improved local control in selected cases [10, 11].

Despite these advances, there remains a paucity of data specifically addressing intraoral desmoplastic melanoma. Reports integrating surgical, reconstructive, and oncologic considerations remain limited.

The objective of this report is to describe a rare case of intraoral desmoplastic melanoma managed with radical composite resection and immediate microvascular reconstruction, and to discuss the associated diagnostic challenges, reconstructive strategy, and key oncologic considerations, including the role of adjuvant therapy and follow-up outcomes.

## Case presentation

A male patient in his fourth decade presented with a progressively enlarging right orofacial mass. Clinical examination revealed a 4-cm exophytic intraoral lesion with deep infiltration involving the upper gingiva, right hemimaxilla, palate, and adjacent musculature. Incisional biopsy confirmed a malignant spindle cell neoplasm consistent with desmoplastic melanoma.

**Figure 1:**
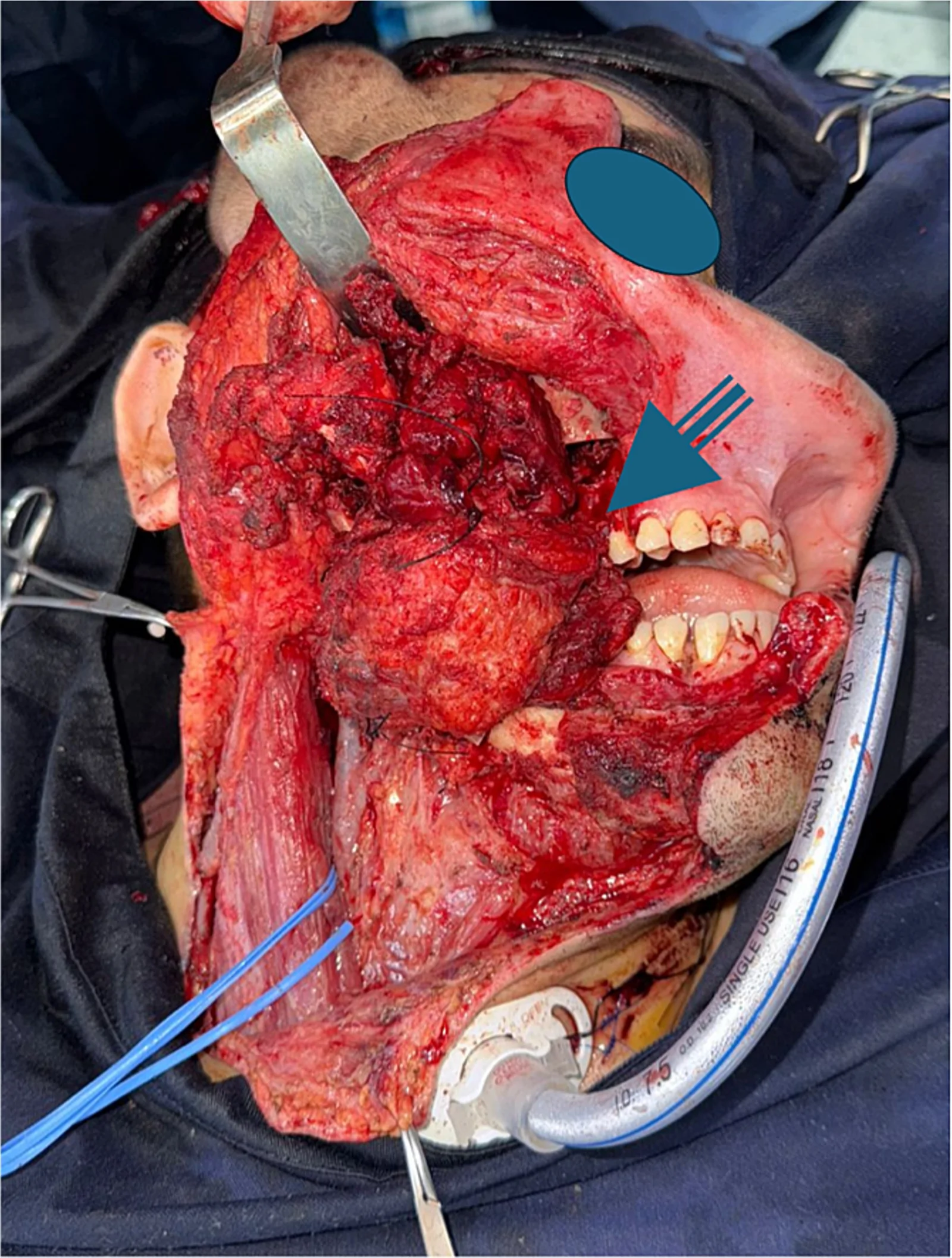
Intraoperative exposure of a large tumor mass involving the right orofacial region, with an exophytic intraoral component and deep extension into the cheek, floor of mouth, and gingivoalveolar complex.

**Figure 2:**
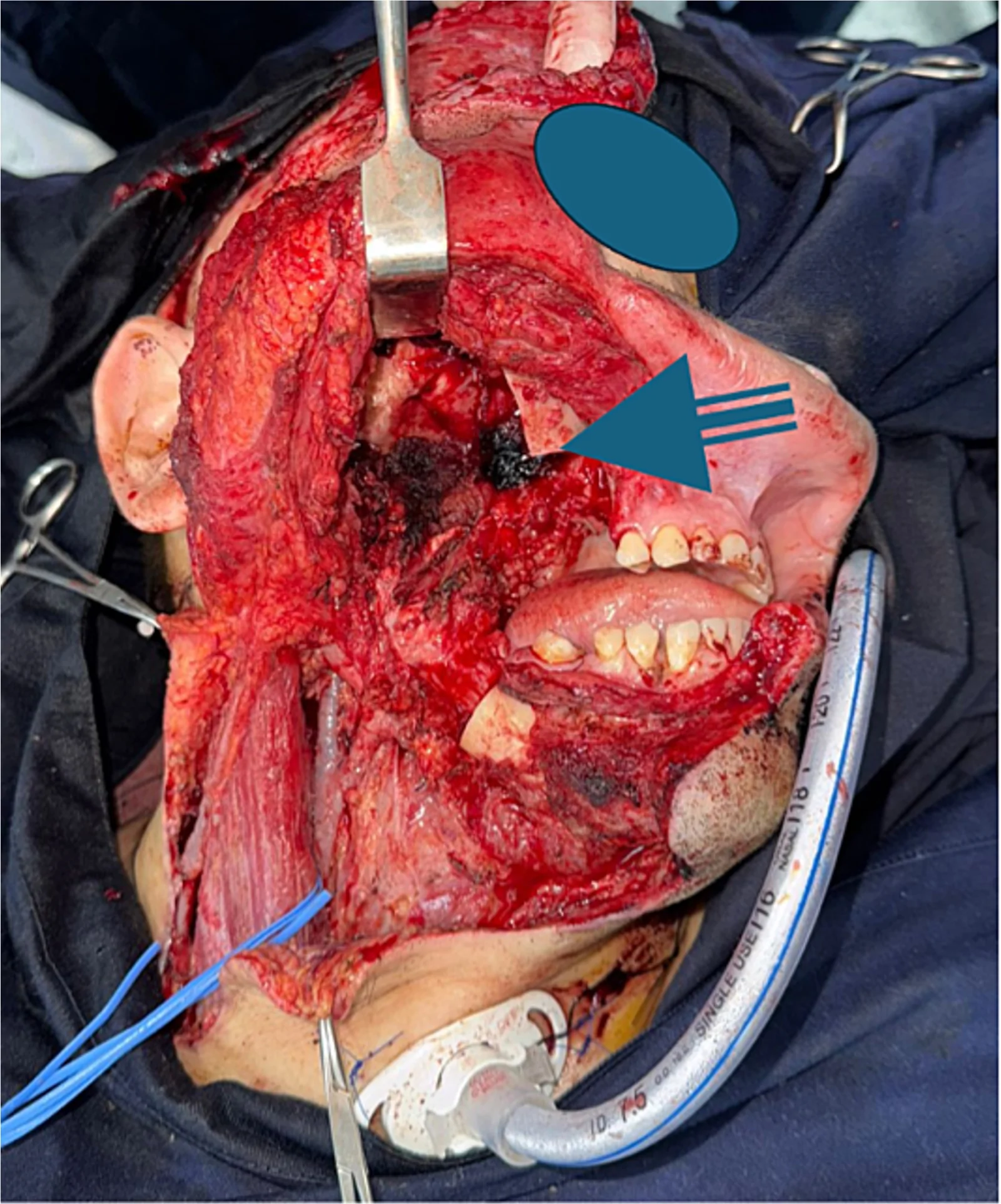
Intraoperative view following wide oncologic resection, demonstrating an extensive three-dimensional composite defect involving the right hemimaxilla and hemimandible, floor of mouth, and cheek, prior to immediate microvascular reconstruction.

Given the extent of local invasion, radical oncologic resection was performed, including hemimaxillectomy, segmental hemimandibulectomy with disarticulation, buccopharyngectomy, and modified radical neck dissection (levels I–V). A transpalatal approach demonstrated tumor extension into the maxillary sinus (Fig. 1). The resulting composite defect involved the oral cavity, midface, mandible, and adjacent soft tissues (Fig. 2).

**Figure 3:**
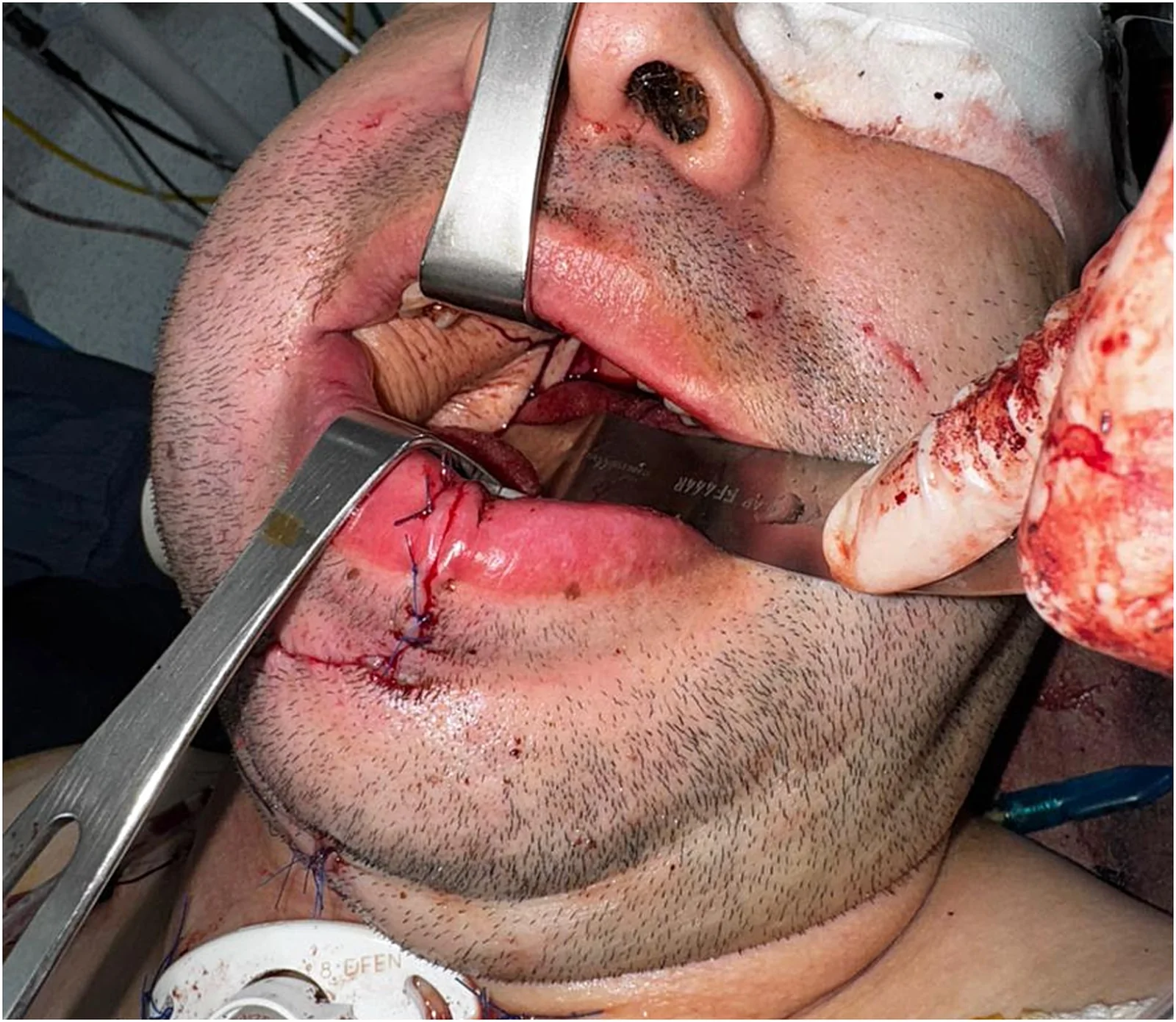
Post-reconstruction intraoral view of the ALT free flap, showing adequate defect coverage and clinical signs of satisfactory perfusion, without evidence of venous congestion or arterial insufficiency.

Immediate reconstruction was performed using a composite ALT free flap. Microvascular anastomoses were established using the facial artery and cervical venous system, achieving adequate perfusion (Fig. 3). The flap was contoured to reconstruct the palate, floor of mouth, cheek, and pharyngeal wall.

Postoperative recovery was uneventful, with no flap-related complications. At 12 months of follow-up, no clinical or radiological evidence of locoregional recurrence was observed. While this represents meaningful short-term outcome data, it remains limited given the risk of late recurrence in mucosal melanoma.

Molecular profiling, including B-Raf proto-oncogene, serine/threonine kinase, KIT proto-oncogene, receptor tyrosine kinase, NRAS proto-oncogene, GTPase, and NF1 mutations, was not performed due to institutional resource constraints. Adjuvant systemic therapy was not administered; however, its indication was formally evaluated within a multidisciplinary tumor board.

**Figure 4:**
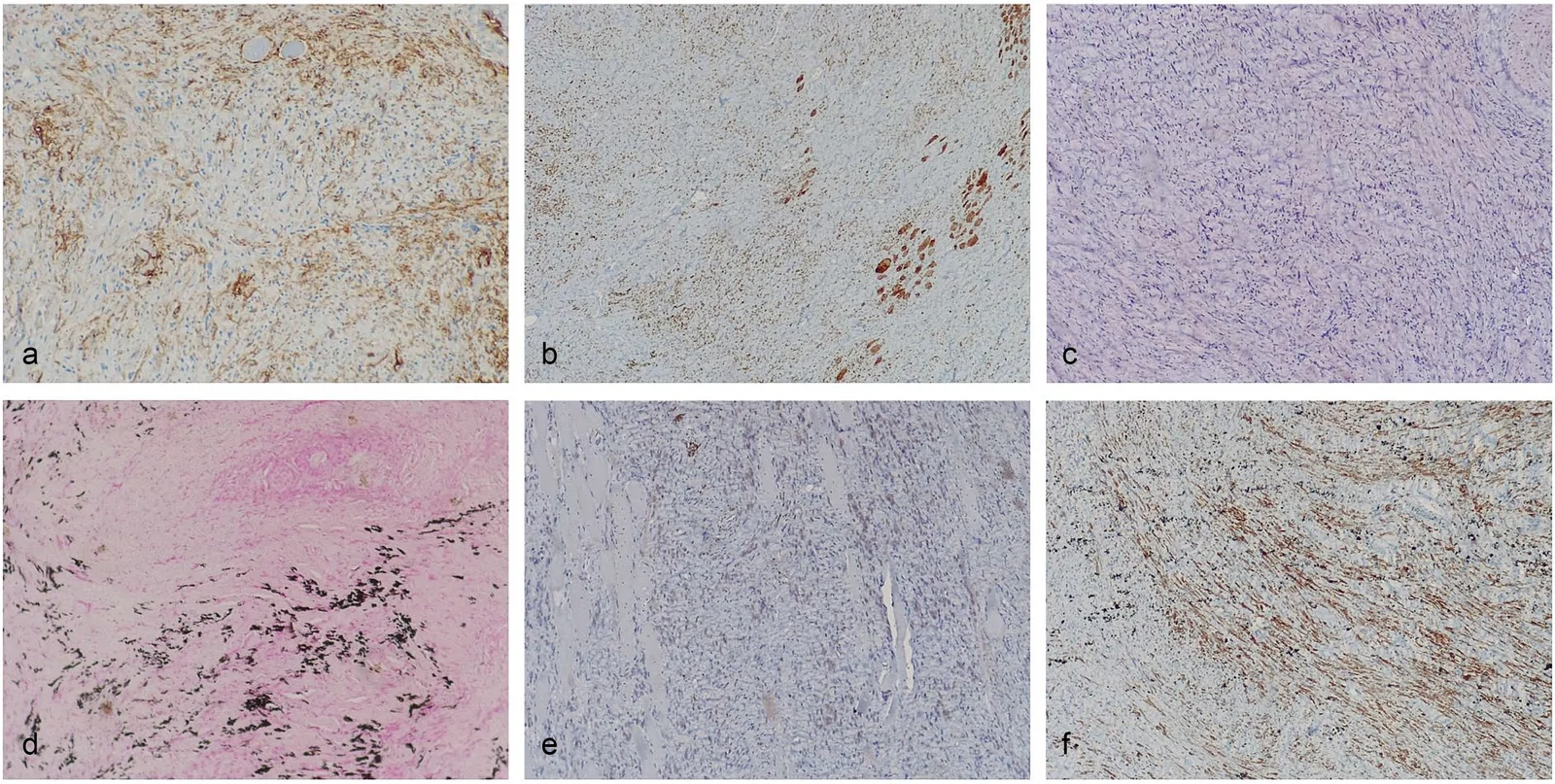
Histopathological evaluation demonstrating spindle cell proliferation arranged in intersecting fascicles within a densely collagenized stroma, consistent with desmoplastic melanoma. (a) CD31 immunohistochemical stain showing endothelial cell positivity within intratumoral vessels; tumor spindle cells are negative (original magnification ×10). (b) CD34 immunohistochemical stain demonstrating diffuse stromal and vascular positivity, with strong expression in tumor spindle cells (original magnification ×10). (c) Desmin immunohistochemical stain showing absence of significant tumor cell expression (original magnification ×10). (d) Fontana–Masson stain highlighting abundant intratumoral melanin pigment (original magnification ×10). (e) Diffuse nuclear SOX10 expression in tumor cells (original magnification ×10). (f) Diffuse cytoplasmic WT1 expression within the spindle cell proliferation in a fibrous/desmoplastic background (original magnification ×10).

Histopathological evaluation demonstrated a spindle cell neoplasm with focal melanin pigment. Immunohistochemistry showed weak S100 positivity and limited SOX10 expression, with strong CD34 positivity and diffuse WT1 nuclear staining (Fig. 4).

## Discussion

Intraoral desmoplastic melanoma is a rare malignancy with significant diagnostic and therapeutic challenges [2, 5]. Its nonspecific clinical presentation and frequent lack of pigmentation contribute to delayed diagnosis [3].

The strong CD34 expression observed in this case represents an unusual finding and raises important differential diagnostic considerations, including solitary fibrous tumor and dermatofibrosarcoma protuberans. However, these entities were excluded based on morphological features, lack of STAT6 expression, and the identification of melanin pigment. CD34 expression was therefore interpreted as nonspecific and possibly related to tumor–stromal interaction [6].

WT1 positivity, although reported in melanocytic lesions, remains nonspecific and should be interpreted cautiously. In this case, it was considered a supportive but non-definitive marker, with diagnosis based on integrated histopathological evaluation.

From an oncologic perspective, desmoplastic melanoma demonstrates a high propensity for local recurrence due to its infiltrative growth pattern and perineural invasion [8]. Wide surgical margins remain essential.

Reconstruction using the ALT free flap enabled restoration of complex three-dimensional defects with favorable functional outcomes [9, 10].

Current evidence supports the use of adjuvant immunotherapy in high-risk melanoma. Anti-PD-1 agents have demonstrated survival benefits, although outcomes in mucosal melanoma are less favorable [11, 12]. In this case, adjuvant therapy was not administered due to limited access, representing an important limitation [13, 14].

Adjuvant radiotherapy may improve local control in desmoplastic melanoma, particularly in high-risk settings [15]. However, data specific to intraoral disease remain limited.

A review of the literature confirms the rarity of intraoral presentations and highlights increasing use of multimodal strategies (Table 1).

**Table 1:** Summary of published cases and series of desmoplastic melanoma.

Authors	Article title	Article type	Year	Country	Cases	Age / Gender	Location	Lesion duration	Size (cm)	Symptoms	Recurrence	Complications
Jain *et al*. [1]	Desmoplastic malignant melanoma and its variants. A study of 45 cases	Retrospective	1989	Australia	45	Range from 42 to 91 years, with a peak in the seventh decade. 31 Male, 14 Female	Head and neck (35 cases), shoulder and arm (four), back and chest (three), abdomen (one), thigh (one) and leg (one).	Not specified	Mean 2.5	Often amelanotic, firm masses	Twenty-three patients (55%) developed one or more local recurrences; 17 (40%) developed distant spread, including extension into the cranial cavity along nerves; and 14 (33%) had both local and distal recurrences.	5 patients (36%) had died of the disease or were terminally ill
Devaraj *et al*. [2]	Desmoplastic Melanoma: A Clinico-Pathological Review	Retrospective	1992	UK	13	8 Female / 5 Male 67 years (range 34–87)	2/3 of the lesions were in the head and neck.	Variable	2–3.5 Tumor thickness averaged 5.78 mm (Breslow).	Variable	7 patients developed recurrence, 4 as regional nodes, and 3 as skin nodules. Four of these patients developed disseminated disease, of whom 3 died.	4 deaths from metastases
Kurihara *et al*. [3]	Desmoplastic Malignant Melanoma of the Gingiva	Case Report	1992	Japan	1	58/ Male	Right maxillary alveolus	~1 year	1.5	Gingival tumor	Local recurrence	Metastasis to neck node
Anstey *et al*. [4]	Desmoplastic Malignant Melanoma: An Immunocytochemical Study of 25 Cases	Retrospective	1993	UK	25	Not detailed	Likely cutaneous	Not specified	Not specified	Not specified	Not specified	Not specified
Carlson *et al*. [5]	Desmoplastic Neurotropic Melanoma: A Clinicopathologic Analysis of 28 Cases	Retrospective	1995	USA	28	Mean and median age, 59 years; range, 22–83 years 15 / Male, 13 / Female	Most tumors were located on the head and neck (75%) and were nonpigmented (57%)	Months to years	Mean depth 4.08 mm	Mostly painless, non-pigmented nodules	Seven patients had recurrent local tumor (multiple in four); four had lymph node metastases, and three had visceral metastases.	Not specified
Kilpatrick *et al*. [6]	Desmoplastic Malignant Melanoma of the Oral Mucosa: An Underrecognized Diagnostic Pitfall	Retrospective	1996	USA	3	42 Male, 64 Male, 75 Male	The left maxillary oral mucosa (two patients) and the vermilion border of the lower lip (one patient)	Months to 2+ years	1.7 × 3.2, 4.5 × 3	Pain, epistaxis, swelling	1 metastasis and death	Misdiagnosis, perineural invasion
Ueta *et al*. [7]	Desmoplastic Malignant Melanoma of the Gingiva: Case Report and Review of the Literature	Case Report	1996	Japan	1	66 / Female	Right molar gingiva	Not specified	3.5 × 2.5	Gingival swelling	This tumor metastasised to the submandibular lymph node 5 years after extirpation, and local recurrence was observed 2 years later.	Misdiagnosis, later recurrence/metastasis
Kavanagh *et al*. [8]	Desmoplastic malignant melanoma of the palatal alveolar mucosa: sustained disease-free survival after surgery and postoperative radiotherapy	Case Report	2000	USA	1	53/ Female	Left palatal mucosa	2 months	~2 × 1	Palatal pain	No	Post-op complications (xerostomia, nasal speech)
Kay *et al.* [9]	Desmoplastic Melanoma of the Head and Neck: Histopathologic and Immunohistochemical Study of 28 Cases	Retrospective	2004	USA	28	Patients averaged 65 years of age. The 17 male (61%) and 11 female (39%)	The cheek was the most common location (12 cases, 43%)	Not specified	Mean 2.8 (0.5–6.0)	Mostly painless nodules	10/21 local recurrence	Not specified
Prasad *et al*. [10]	Primary Mucosal Desmoplastic Melanoma of the Head and Neck	Retrospective	2004	USA	7	(23–74 years) 6 Male, 1 Female	Desmoplastic melanoma involved the lip in two, alveolus in three, buccal mucosa in one, and nasal vestibule in one patient	2–12 months	0.6–2.0	Ulcer, mass, Bell’s palsy	7/7 (100%)	Six patients died with disease (survival, 1–41 years; median, 8 years), and one patient was free of disease (survival 20 years)
Ramani *et al*. [11]	Desmoplastic Malignant Melanoma of Alveolus – A Rare Entity	Case Report	2006	India	1	32 / Male	Left posterior maxillary alveolus	20 days	4 × 5	Pain, swelling	Not specified	No
Gru *et al*. [12]	Mucosal melanoma: correlation of clinicopathologic, prognostic, and molecular features	Clinicopathologic & molecular correlation	2014	USA	84 patients, 132 specimens	Not individually detailed	Head & neck mucosal sites (oral, sinonasal, gynecologic, etc.)	Not reported	> 3 cm vs ≤ 3 cm associated survival difference	-	-	Head and neck tumors >3 cm: mean survival ~12.8 months; ≤3 cm: ~38.3 months (P = 0.035). Pure epithelioid histology: mean survival 16.8 months (P = 0.028). Spindle morphology fared better. EWSR1 rearrangement was rare. Multivariate analysis confirmed size and histology as independent prognostic factors
Smith *et al*. [13]	Primary Sinonasal Mucosal Melanoma with Aberrant Diffuse and Strong Desmin Reactivity: A Potential Diagnostic Pitfall!	Case report	2015	USA	1	70/ Male	Sphenoid sinus (sinonasal tract)	Not reported	Not reported	Not specified	Not specified	Initial misdiagnosis as rhabdomyosarcoma due to desmin positivity; finally diagnosed as melanoma on IHC
Min *et al*. [14]	Desmoplastic melanoma of the oral cavity: diagnostic pitfalls and clinical characteristics	Original article	2018	South Korea	1 (plus 19 literature cases)	74 / Female	Hard palate	2 months	1.8 × 1.5	Ulceration	None at 12 months	Oroantral fistula (closed)
Ortega *et al*. [15]	Nasal Mucosal Desmoplastic Melanoma: A Case Report with Review of the Literature	Case report + review	2022	EE.UU. (Tennessee)	1	79/ Female	Nasal vestibule	Not reported	0.3–0.4 cm lesion on the lateral wall of the right nasal vestibule	Nasal discomfort, rhinorrhea	Not reported	Initial excision with positive margins; re-resection showed no residual tumor

## Conclusion

Intraoral desmoplastic melanoma is a rare and diagnostically complex malignancy that requires a high index of suspicion and careful histopathological evaluation.

Radical surgical resection combined with microvascular reconstruction allows effective local control and functional restoration. However, optimal management extends beyond surgery and should include multidisciplinary evaluation and consideration of adjuvant therapies when feasible. Limitations such as absence of molecular profiling and relatively short follow-up should be acknowledged. Long-term surveillance and individualized treatment strategies remain essential to optimize outcomes.
